# Ethyl 3-bromo-1-(3-chloro-2-pyrid­yl)-4,5-dihydro-1*H*-pyrazole-5-carboxyl­ate

**DOI:** 10.1107/S1600536809050673

**Published:** 2009-11-28

**Authors:** Hua Chen, Huibin Yang, Haibo Yu, Bin Li

**Affiliations:** a Shenyang Institute of Chemical Technology, Shenyang 110142, People’s Republic of China; b Agrochemicals Division, Shenyang Research Institute of Chemical Industry, Shenyang 110021, People’s Republic of China

## Abstract

The title compound, C_11_H_11_BrClN_3_O_2_, contains two mol­ecules in the asymmetric unit in which the dihedral angles between the pyrazole and pyridine rings are 30.0 (2) and 22.3 (2)°.

## Related literature

For background to the use of anthranilamide compounds containing *N*-pyridyl pyrazole groups as potential insecticides, see: Lahm *et al.* (2003 ▶). For the synthesis, see: Lahm *et al.* (2005 ▶).
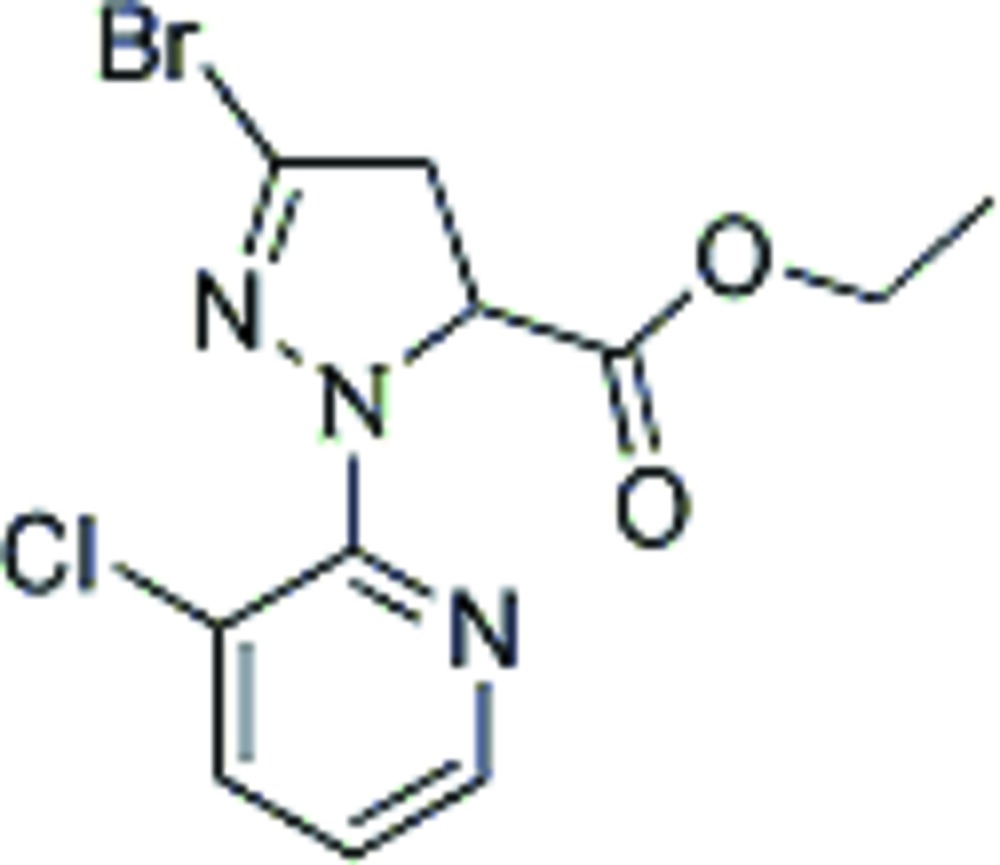


## Experimental

### 

#### Crystal data

C_11_H_11_BrClN_3_O_2_
*M* *_r_* = 332.59
Monoclinic, 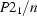

*a* = 11.9977 (18) Å
*b* = 10.8520 (17) Å
*c* = 20.762 (3) Å
β = 93.388 (3)°
*V* = 2698.4 (7) Å^3^
*Z* = 8
Mo *K*α radiation
μ = 3.24 mm^−1^
*T* = 296 K
0.38 × 0.32 × 0.30 mm

#### Data collection

Bruker SMART CCD diffractometer
Absorption correction: multi-scan (*SADABS*; Bruker, 2001 ▶) *T*_min_ = 0.356, *T*_max_ = 1.000
13418 measured reflections
4760 independent reflections
2823 reflections with *I* > 2σ(*I*)
*R*_int_ = 0.029

#### Refinement

*R*[*F* ^2^ > 2σ(*F* ^2^)] = 0.042
*wR*(*F* ^2^) = 0.126
*S* = 1.02
4760 reflections
327 parameters
H-atom parameters constrained
Δρ_max_ = 0.64 e Å^−3^
Δρ_min_ = −0.49 e Å^−3^

### 

Data collection: *SMART* (Bruker, 2001 ▶); cell refinement: *SAINT* (Bruker, 2001 ▶); data reduction: *SAINT*; program(s) used to solve structure: *SHELXS97* (Sheldrick, 2008 ▶); program(s) used to refine structure: *SHELXL97* (Sheldrick, 2008 ▶); molecular graphics: *SHELXTL* (Sheldrick, 2008 ▶); software used to prepare material for publication: *SHELXTL*.

## Supplementary Material

Crystal structure: contains datablocks I, global. DOI: 10.1107/S1600536809050673/hb5251sup1.cif

Structure factors: contains datablocks I. DOI: 10.1107/S1600536809050673/hb5251Isup2.hkl

Additional supplementary materials:  crystallographic information; 3D view; checkCIF report

## Figures and Tables

**Fig. 1.**
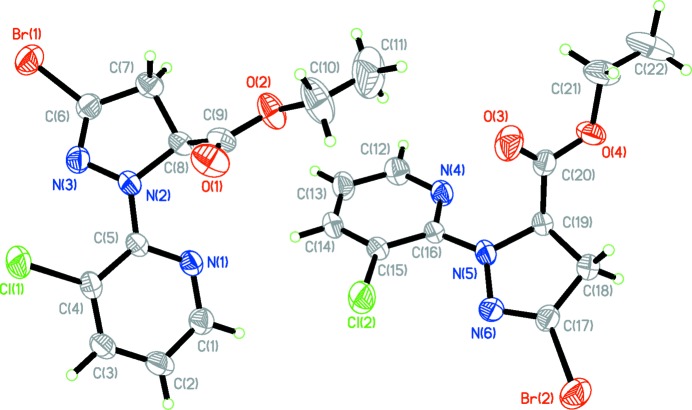
The molecular structure of (I), with 30% probability displacement ellipsoids.

## supplementary crystallographic information

### Experimental

According to the reported procedure of Lahm *et al.*, (2005), the title compound was synthesized by ethyl 2-(3-chloro-2-Pyridinyl)-5-oxo-3-pyrazolidinecarboxylate with phosphorus oxybromide under basic condition in acetonitrile. The crude products were purified by silica-gel column chromatography and then grown from acetone to afford colorless single crystals suitable for X-ray diffraction.To a solution of ethyl 2-(3-chloro-2-Pyridinyl)-5-oxo-3-pyrazolidinecarboxylate (2.70 g, 10.0 mmol) in acetonitrile (30 ml) was added the solution of phosphorusoxybromide (2.01 g, 7.0 mmol) in acetonitrile (30 ml) at 333 K.The reaction mixturewas heated to reflux at 369 K over period of 1 h. Then neutralize concentrated reaction mixture with sodium bicarbonate until the PH=8.Then the mixture was extracted with ethyl acetate; the organic extracts were dried overmagnesium sulfate and concentrated. Afford the title product compound as a white solid (2.5 g, 75%). Anal. Calcd for C_11_H_11_N_3_O_2_: C, 39.72; H, 3.33; N, 12.63. Found: C, 39.91; H, 3.27; N, 12.50. ^1^H NMR (DMSO): 1.15(t, 3H, CH_3_), 3.29 (dd, 1H, pyrazole-H), 3.60 (dd, 1H, pyrazole-H),4.11(q,2*H*,CH_2_),5.20 (dd, 1H, pyrazole-H), 6.99 (dd, 1H, pyridine-H), 7.84 (d, 1H, pyridine-H), 8.12 (d, 1H, pyridine-H).

### Refinement

All H atoms were visible in difference maps: they were placed in geometrically calculated positions, with C—H = 0.93–0.98 Å, and refined as riding with *U*_iso_(H) = 1.2*U*_eq_(C) and *U*_iso_(H) = 1.5*U*_eq_(methyl C).

### Crystal data

**Table:**

C_11_H_11_BrClN_3_O_2_	*F*(000) = 1328
*M**_r_* = 332.59	*D*_x_ = 1.637 Mg m^−^^3^
Monoclinic, *P*2_1_/*n*	Mo *K*α radiation, λ = 0.71073 Å
Hall symbol: -P 2yn	Cell parameters from 2849 reflections
*a* = 11.9977 (18) Å	θ = 2.5–23.4°
*b* = 10.8520 (17) Å	µ = 3.24 mm^−^^1^
*c* = 20.762 (3) Å	*T* = 296 K
β = 93.388 (3)°	Block, colourless
*V* = 2698.4 (7) Å^3^	0.38 × 0.32 × 0.30 mm
*Z* = 8	

### Data collection

**Table:**

Bruker SMART CCD diffractometer	4760 independent reflections
Radiation source: fine-focus sealed tube	2823 reflections with *I* > 2σ(*I*)
graphite	*R*_int_ = 0.029
ω scans	θ_max_ = 25.0°, θ_min_ = 1.9°
Absorption correction: multi-scan (*SADABS*; Bruker, 2001)	*h* = −14→14
*T*_min_ = 0.356, *T*_max_ = 1.000	*k* = −12→8
13418 measured reflections	*l* = −20→24

### Refinement

**Table:**

Refinement on *F*^2^	Primary atom site location: structure-invariant direct methods
Least-squares matrix: full	Secondary atom site location: difference Fourier map
*R*[*F*^2^ > 2σ(*F*^2^)] = 0.042	Hydrogen site location: inferred from neighbouring sites
*wR*(*F*^2^) = 0.126	H-atom parameters constrained
*S* = 1.02	*w* = 1/[σ^2^(*F*_o_^2^) + (0.0609*P*)^2^ + 0.7833*P*] where *P* = (*F*_o_^2^ + 2*F*_c_^2^)/3
4760 reflections	(Δ/σ)_max_ = 0.001
327 parameters	Δρ_max_ = 0.64 e Å^−^^3^
0 restraints	Δρ_min_ = −0.49 e Å^−^^3^

### Special details

**Table:**

Geometry. All e.s.d.'s (except the e.s.d. in the dihedral angle between two l.s. planes) are estimated using the full covariance matrix. The cell e.s.d.'s are taken into account individually in the estimation of e.s.d.'s in distances, angles and torsion angles; correlations between e.s.d.'s in cell parameters are only used when they are defined by crystal symmetry. An approximate (isotropic) treatment of cell e.s.d.'s is used for estimating e.s.d.'s involving l.s. planes.
Refinement. Refinement of *F*^2^ against ALL reflections. The weighted *R*-factor *wR* and goodness of fit *S* are based on *F*^2^, conventional *R*-factors *R* are based on *F*, with *F* set to zero for negative *F*^2^. The threshold expression of *F*^2^ > σ(*F*^2^) is used only for calculating *R*-factors(gt) *etc*. and is not relevant to the choice of reflections for refinement. *R*-factors based on *F*^2^ are statistically about twice as large as those based on *F*, and *R*- factors based on ALL data will be even larger.

### Fractional atomic coordinates and isotropic or equivalent isotropic displacement parameters (Å^2^)

**Table:**

	*x*	*y*	*z*	*U*_iso_*/*U*_eq_	
Br1	0.61938 (4)	0.12589 (5)	0.11537 (3)	0.0919 (2)	
Br2	0.12166 (4)	1.34462 (4)	0.11712 (3)	0.0871 (2)	
Cl1	0.84050 (8)	0.53502 (11)	0.12056 (6)	0.0843 (4)	
Cl2	0.33983 (8)	0.94532 (12)	0.10673 (7)	0.0940 (4)	
O1	0.4918 (3)	0.6080 (3)	0.20858 (15)	0.0867 (10)	
O2	0.3214 (2)	0.5466 (3)	0.17635 (13)	0.0802 (9)	
O3	0.0011 (3)	0.8736 (3)	0.20050 (14)	0.0864 (10)	
O4	−0.1747 (2)	0.9229 (3)	0.17066 (12)	0.0680 (8)	
N1	0.5454 (2)	0.6660 (3)	0.05716 (14)	0.0553 (8)	
N2	0.5862 (2)	0.4848 (3)	0.11040 (15)	0.0610 (9)	
N3	0.6393 (3)	0.3738 (3)	0.10056 (16)	0.0601 (9)	
N4	0.0445 (2)	0.8001 (3)	0.05316 (15)	0.0577 (8)	
N5	0.0807 (2)	0.9898 (3)	0.09552 (19)	0.0750 (10)	
N6	0.1371 (2)	1.0989 (3)	0.09295 (15)	0.0579 (8)	
C1	0.5772 (3)	0.7712 (4)	0.03040 (18)	0.0640 (11)	
H1	0.5218	0.8257	0.0153	0.077*	
C2	0.6850 (3)	0.8036 (4)	0.02391 (19)	0.0700 (12)	
H2	0.7031	0.8752	0.0023	0.084*	
C3	0.7664 (3)	0.7272 (4)	0.05023 (19)	0.0619 (11)	
H3	0.8413	0.7475	0.0475	0.074*	
C4	0.7373 (3)	0.6211 (4)	0.08063 (18)	0.0548 (10)	
C5	0.6239 (3)	0.5907 (3)	0.08114 (16)	0.0477 (9)	
C6	0.5731 (3)	0.2893 (4)	0.11589 (18)	0.0595 (10)	
C7	0.4634 (3)	0.3298 (4)	0.13789 (19)	0.0610 (10)	
H7A	0.4583	0.3175	0.1839	0.073*	
H7B	0.4019	0.2878	0.1147	0.073*	
C8	0.4669 (3)	0.4674 (3)	0.12040 (17)	0.0528 (9)	
H8	0.4219	0.4833	0.0803	0.063*	
C9	0.4312 (3)	0.5498 (4)	0.17334 (18)	0.0592 (10)	
C10	0.2759 (5)	0.6186 (6)	0.2280 (3)	0.119 (2)	
H10A	0.3045	0.5872	0.2695	0.143*	
H10B	0.2990	0.7039	0.2246	0.143*	
C11	0.1599 (6)	0.6115 (7)	0.2238 (3)	0.159 (3)	
H11A	0.1319	0.6407	0.1823	0.238*	
H11B	0.1303	0.6614	0.2569	0.238*	
H11C	0.1373	0.5274	0.2292	0.238*	
C12	0.0767 (3)	0.6904 (4)	0.03225 (19)	0.0664 (11)	
H12	0.0218	0.6335	0.0197	0.080*	
C13	0.1851 (3)	0.6570 (4)	0.0282 (2)	0.0708 (12)	
H13	0.2042	0.5807	0.0117	0.085*	
C14	0.2656 (3)	0.7401 (4)	0.0494 (2)	0.0658 (11)	
H14	0.3407	0.7203	0.0474	0.079*	
C15	0.2355 (3)	0.8510 (4)	0.07329 (19)	0.0556 (10)	
C16	0.1219 (3)	0.8812 (3)	0.07301 (17)	0.0490 (9)	
C17	0.0712 (3)	1.1825 (4)	0.10999 (18)	0.0561 (10)	
C18	−0.0415 (3)	1.1433 (4)	0.1270 (2)	0.0679 (12)	
H18A	−0.0997	1.1861	0.1013	0.081*	
H18B	−0.0524	1.1557	0.1724	0.081*	
C19	−0.0372 (3)	1.0054 (3)	0.10966 (17)	0.0534 (9)	
H19	−0.0863	0.9873	0.0715	0.064*	
C20	−0.0653 (3)	0.9256 (4)	0.16513 (18)	0.0551 (10)	
C21	−0.2137 (5)	0.8554 (5)	0.2256 (2)	0.0970 (17)	
H21A	−0.1850	0.7719	0.2256	0.116*	
H21B	−0.1870	0.8950	0.2654	0.116*	
C22	−0.3344 (5)	0.8532 (6)	0.2213 (3)	0.133 (3)	
H22A	−0.3623	0.9361	0.2203	0.199*	
H22B	−0.3606	0.8109	0.2581	0.199*	
H22C	−0.3603	0.8111	0.1826	0.199*	

### Atomic displacement parameters (Å^2^)

**Table:**

	*U*^11^	*U*^22^	*U*^33^	*U*^12^	*U*^13^	*U*^23^
Br1	0.0860 (4)	0.0580 (3)	0.1307 (5)	0.0064 (2)	−0.0007 (3)	0.0112 (3)
Br2	0.0843 (4)	0.0538 (3)	0.1226 (4)	−0.0033 (2)	0.0012 (3)	−0.0117 (3)
Cl1	0.0455 (6)	0.0807 (8)	0.1243 (10)	0.0010 (5)	−0.0147 (6)	0.0073 (7)
Cl2	0.0447 (6)	0.0696 (8)	0.1656 (12)	−0.0029 (5)	−0.0116 (7)	−0.0069 (8)
O1	0.086 (2)	0.093 (2)	0.080 (2)	−0.0312 (19)	0.0031 (17)	−0.0221 (18)
O2	0.0583 (18)	0.104 (3)	0.0802 (19)	−0.0035 (16)	0.0172 (14)	−0.0382 (18)
O3	0.086 (2)	0.096 (3)	0.077 (2)	0.0324 (18)	−0.0057 (17)	0.0147 (17)
O4	0.0564 (17)	0.084 (2)	0.0646 (17)	−0.0057 (14)	0.0118 (13)	0.0251 (15)
N1	0.0409 (17)	0.063 (2)	0.061 (2)	−0.0002 (16)	0.0015 (14)	0.0043 (17)
N2	0.0402 (17)	0.056 (2)	0.088 (2)	−0.0019 (15)	0.0116 (15)	0.0054 (18)
N3	0.0498 (19)	0.057 (2)	0.073 (2)	0.0037 (16)	−0.0023 (16)	0.0003 (17)
N4	0.0416 (17)	0.062 (2)	0.070 (2)	0.0016 (16)	0.0021 (15)	−0.0019 (17)
N5	0.0445 (19)	0.051 (2)	0.132 (3)	0.0018 (16)	0.0298 (19)	−0.002 (2)
N6	0.0467 (18)	0.050 (2)	0.077 (2)	−0.0038 (16)	0.0032 (15)	0.0022 (17)
C1	0.055 (2)	0.071 (3)	0.065 (3)	0.005 (2)	0.0026 (19)	0.010 (2)
C2	0.060 (3)	0.077 (3)	0.074 (3)	−0.010 (2)	0.016 (2)	0.015 (2)
C3	0.048 (2)	0.065 (3)	0.074 (3)	−0.009 (2)	0.0169 (19)	−0.007 (2)
C4	0.040 (2)	0.061 (3)	0.064 (2)	0.0042 (18)	0.0026 (17)	−0.007 (2)
C5	0.039 (2)	0.052 (2)	0.053 (2)	−0.0038 (17)	0.0079 (16)	−0.0054 (18)
C6	0.054 (2)	0.056 (3)	0.067 (3)	−0.003 (2)	−0.0088 (19)	0.002 (2)
C7	0.059 (2)	0.060 (3)	0.065 (3)	−0.012 (2)	0.0060 (19)	−0.009 (2)
C8	0.043 (2)	0.059 (3)	0.056 (2)	−0.0105 (17)	0.0055 (16)	−0.0042 (19)
C9	0.055 (2)	0.062 (3)	0.060 (2)	−0.016 (2)	0.004 (2)	−0.002 (2)
C10	0.086 (4)	0.155 (6)	0.120 (5)	0.000 (4)	0.034 (3)	−0.070 (4)
C11	0.172 (7)	0.171 (7)	0.137 (6)	0.073 (6)	0.047 (5)	−0.040 (5)
C12	0.055 (2)	0.071 (3)	0.073 (3)	−0.006 (2)	−0.001 (2)	−0.015 (2)
C13	0.060 (3)	0.067 (3)	0.087 (3)	0.009 (2)	0.018 (2)	−0.015 (2)
C14	0.046 (2)	0.064 (3)	0.089 (3)	0.009 (2)	0.021 (2)	0.000 (2)
C15	0.039 (2)	0.053 (2)	0.075 (3)	−0.0008 (17)	0.0065 (18)	0.007 (2)
C16	0.040 (2)	0.050 (2)	0.057 (2)	0.0047 (17)	0.0104 (17)	0.0067 (18)
C17	0.055 (2)	0.049 (2)	0.064 (3)	0.0074 (19)	0.0002 (18)	0.003 (2)
C18	0.060 (3)	0.057 (3)	0.089 (3)	0.013 (2)	0.021 (2)	0.014 (2)
C19	0.043 (2)	0.052 (2)	0.066 (2)	0.0070 (17)	0.0114 (17)	0.0065 (19)
C20	0.051 (2)	0.050 (2)	0.064 (3)	0.0070 (19)	0.001 (2)	−0.002 (2)
C21	0.111 (4)	0.106 (4)	0.077 (3)	−0.013 (3)	0.030 (3)	0.032 (3)
C22	0.138 (6)	0.166 (7)	0.100 (4)	−0.078 (5)	0.042 (4)	0.012 (4)

### Geometric parameters (Å, °)

**Table:**

Br1—C6	1.858 (4)	C7—C8	1.538 (5)
Br2—C17	1.863 (4)	C7—H7A	0.9700
Cl1—C4	1.724 (4)	C7—H7B	0.9700
Cl2—C15	1.731 (4)	C8—C9	1.499 (5)
O1—C9	1.184 (4)	C8—H8	0.9800
O2—C9	1.323 (4)	C10—C11	1.392 (9)
O2—C10	1.459 (5)	C10—H10A	0.9700
O3—C20	1.192 (4)	C10—H10B	0.9700
O4—C20	1.325 (4)	C11—H11A	0.9600
O4—C21	1.455 (5)	C11—H11B	0.9600
N1—C5	1.321 (4)	C11—H11C	0.9600
N1—C1	1.335 (5)	C12—C13	1.357 (5)
N2—N3	1.383 (4)	C12—H12	0.9300
N2—C5	1.388 (5)	C13—C14	1.374 (6)
N2—C8	1.471 (4)	C13—H13	0.9300
N3—C6	1.266 (5)	C14—C15	1.358 (5)
N4—C16	1.327 (5)	C14—H14	0.9300
N4—C12	1.332 (5)	C15—C16	1.401 (5)
N5—N6	1.366 (4)	C17—C18	1.480 (5)
N5—C16	1.371 (5)	C18—C19	1.541 (5)
N5—C19	1.470 (4)	C18—H18A	0.9700
N6—C17	1.267 (5)	C18—H18B	0.9700
C1—C2	1.355 (5)	C19—C20	1.495 (5)
C1—H1	0.9300	C19—H19	0.9800
C2—C3	1.370 (5)	C21—C22	1.445 (8)
C2—H2	0.9300	C21—H21A	0.9700
C3—C4	1.368 (5)	C21—H21B	0.9700
C3—H3	0.9300	C22—H22A	0.9600
C4—C5	1.401 (5)	C22—H22B	0.9600
C6—C7	1.486 (5)	C22—H22C	0.9600
			
C9—O2—C10	115.9 (3)	C10—C11—H11A	109.5
C20—O4—C21	116.4 (3)	C10—C11—H11B	109.5
C5—N1—C1	118.1 (3)	H11A—C11—H11B	109.5
N3—N2—C5	119.5 (3)	C10—C11—H11C	109.5
N3—N2—C8	111.7 (3)	H11A—C11—H11C	109.5
C5—N2—C8	120.8 (3)	H11B—C11—H11C	109.5
C6—N3—N2	107.0 (3)	N4—C12—C13	123.8 (4)
C16—N4—C12	118.8 (3)	N4—C12—H12	118.1
N6—N5—C16	122.8 (3)	C13—C12—H12	118.1
N6—N5—C19	113.2 (3)	C12—C13—C14	117.5 (4)
C16—N5—C19	122.5 (3)	C12—C13—H13	121.2
C17—N6—N5	106.9 (3)	C14—C13—H13	121.2
N1—C1—C2	124.1 (4)	C15—C14—C13	120.1 (4)
N1—C1—H1	118.0	C15—C14—H14	120.0
C2—C1—H1	118.0	C13—C14—H14	120.0
C1—C2—C3	117.8 (4)	C14—C15—C16	119.0 (4)
C1—C2—H2	121.1	C14—C15—Cl2	118.0 (3)
C3—C2—H2	121.1	C16—C15—Cl2	122.9 (3)
C4—C3—C2	119.8 (4)	N4—C16—N5	114.6 (3)
C4—C3—H3	120.1	N4—C16—C15	120.6 (3)
C2—C3—H3	120.1	N5—C16—C15	124.8 (3)
C3—C4—C5	118.5 (3)	N6—C17—C18	117.0 (4)
C3—C4—Cl1	118.8 (3)	N6—C17—Br2	119.6 (3)
C5—C4—Cl1	122.6 (3)	C18—C17—Br2	123.3 (3)
N1—C5—N2	115.7 (3)	C17—C18—C19	100.3 (3)
N1—C5—C4	121.4 (3)	C17—C18—H18A	111.7
N2—C5—C4	122.7 (3)	C19—C18—H18A	111.7
N3—C6—C7	116.3 (4)	C17—C18—H18B	111.7
N3—C6—Br1	119.9 (3)	C19—C18—H18B	111.7
C7—C6—Br1	123.7 (3)	H18A—C18—H18B	109.5
C6—C7—C8	100.2 (3)	N5—C19—C20	110.5 (3)
C6—C7—H7A	111.7	N5—C19—C18	101.8 (3)
C8—C7—H7A	111.7	C20—C19—C18	111.7 (3)
C6—C7—H7B	111.7	N5—C19—H19	110.8
C8—C7—H7B	111.7	C20—C19—H19	110.8
H7A—C7—H7B	109.5	C18—C19—H19	110.8
N2—C8—C9	110.5 (3)	O3—C20—O4	124.4 (4)
N2—C8—C7	101.4 (3)	O3—C20—C19	125.2 (4)
C9—C8—C7	113.2 (3)	O4—C20—C19	110.4 (3)
N2—C8—H8	110.5	C22—C21—O4	109.1 (4)
C9—C8—H8	110.5	C22—C21—H21A	109.9
C7—C8—H8	110.5	O4—C21—H21A	109.9
O1—C9—O2	124.2 (4)	C22—C21—H21B	109.9
O1—C9—C8	125.4 (4)	O4—C21—H21B	109.9
O2—C9—C8	110.3 (3)	H21A—C21—H21B	108.3
C11—C10—O2	109.9 (5)	C21—C22—H22A	109.5
C11—C10—H10A	109.7	C21—C22—H22B	109.5
O2—C10—H10A	109.7	H22A—C22—H22B	109.5
C11—C10—H10B	109.7	C21—C22—H22C	109.5
O2—C10—H10B	109.7	H22A—C22—H22C	109.5
H10A—C10—H10B	108.2	H22B—C22—H22C	109.5
			
C5—N2—N3—C6	−161.8 (3)	C7—C8—C9—O2	−75.6 (4)
C8—N2—N3—C6	−12.7 (4)	C9—O2—C10—C11	177.3 (5)
C16—N5—N6—C17	172.2 (4)	C16—N4—C12—C13	−1.9 (6)
C19—N5—N6—C17	6.1 (4)	N4—C12—C13—C14	2.6 (7)
C5—N1—C1—C2	2.8 (6)	C12—C13—C14—C15	0.0 (6)
N1—C1—C2—C3	−4.5 (6)	C13—C14—C15—C16	−3.0 (6)
C1—C2—C3—C4	1.3 (6)	C13—C14—C15—Cl2	173.6 (3)
C2—C3—C4—C5	3.0 (6)	C12—N4—C16—N5	−178.0 (4)
C2—C3—C4—Cl1	−173.3 (3)	C12—N4—C16—C15	−1.4 (5)
C1—N1—C5—N2	177.8 (3)	N6—N5—C16—N4	−152.2 (4)
C1—N1—C5—C4	2.0 (5)	C19—N5—C16—N4	12.6 (5)
N3—N2—C5—N1	139.1 (3)	N6—N5—C16—C15	31.3 (6)
C8—N2—C5—N1	−7.1 (5)	C19—N5—C16—C15	−163.9 (3)
N3—N2—C5—C4	−45.1 (5)	C14—C15—C16—N4	3.8 (6)
C8—N2—C5—C4	168.6 (3)	Cl2—C15—C16—N4	−172.6 (3)
C3—C4—C5—N1	−4.9 (5)	C14—C15—C16—N5	−179.9 (4)
Cl1—C4—C5—N1	171.4 (3)	Cl2—C15—C16—N5	3.7 (6)
C3—C4—C5—N2	179.7 (3)	N5—N6—C17—C18	0.0 (5)
Cl1—C4—C5—N2	−4.1 (5)	N5—N6—C17—Br2	176.5 (3)
N2—N3—C6—C7	0.8 (5)	N6—C17—C18—C19	−5.4 (5)
N2—N3—C6—Br1	−175.1 (2)	Br2—C17—C18—C19	178.2 (3)
N3—C6—C7—C8	10.3 (4)	N6—N5—C19—C20	−127.9 (4)
Br1—C6—C7—C8	−173.9 (3)	C16—N5—C19—C20	66.0 (5)
N3—N2—C8—C9	138.6 (3)	N6—N5—C19—C18	−9.0 (4)
C5—N2—C8—C9	−72.8 (4)	C16—N5—C19—C18	−175.2 (4)
N3—N2—C8—C7	18.3 (4)	C17—C18—C19—N5	7.8 (4)
C5—N2—C8—C7	166.9 (3)	C17—C18—C19—C20	125.8 (3)
C6—C7—C8—N2	−15.7 (3)	C21—O4—C20—O3	2.7 (6)
C6—C7—C8—C9	−134.1 (3)	C21—O4—C20—C19	−175.9 (4)
C10—O2—C9—O1	−1.4 (7)	N5—C19—C20—O3	12.4 (5)
C10—O2—C9—C8	177.2 (4)	C18—C19—C20—O3	−100.1 (5)
N2—C8—C9—O1	−9.9 (6)	N5—C19—C20—O4	−169.0 (3)
C7—C8—C9—O1	103.0 (5)	C18—C19—C20—O4	78.4 (4)
N2—C8—C9—O2	171.5 (3)	C20—O4—C21—C22	−175.3 (4)

## References

[bb1] Bruker (2001). *SMART*, *SAINT* and *SADABS*. Bruker AXS Inc., Madison, Wisconsin, USA.

[bb2] Lahm, G. P., Selby, T. P. & Freudenberger, J. H. (2005). *Bioorg. Med. Chem. Lett.* **15**, 4898–4906. 10.1016/j.bmcl.2005.08.034 16165355

[bb3] Lahm, G. P., Selby, T. P. & Stevenson, T. M. (2003). World Patent No. WO 03/015519.

[bb4] Sheldrick, G. M. (2008). *Acta Cryst.* A**64**, 112–122. 10.1107/S0108767307043930 18156677

